# Supplementary material for: Evaluation of the Potency of Anti-HIV and Anti-HCV Drugs to Inhibit P-Glycoprotein Mediated Efflux of Digoxin in Caco-2 Cell Line and Human Precision-Cut Intestinal Slices
Source: Pharmaceuticals (Basel). 2022 Feb 18;15(2):242. doi: 10.3390/ph15020242 (PMC8875242; doi:10.3390/ph15020242)
Supplement: Supplementary file 1 [file pharmaceuticals-15-00242-s001.zip › pharmaceuticals-1573074-supplementary.pdf]

SUPPLEMENTARY MATERIAL

Evaluation of the Potency of Anti-HIV and Anti-HCV Drugs to Inhibit P-Glycoprotein Mediated Efflux of Digoxin in Caco-2 Cell Line and Human Precision-Cut Intestinal Slices

Martin Huličiak [1], Ivan Vokřál [1,*], Ondřej Holas [2], Ondřej Martinec [1], František Štaud [1] and Lukáš Červený [1]

[1] Department of Pharmacology and Toxicology, Faculty of Pharmacy in Hradec Králové, Charles University, 50005 Hradec Králové, Czech Republic; hulicim@faf.cuni.cz (M.H.); francuk@gmail.com (O.M.); staud@faf.cuni.cz (F.Š.); cervenyl@faf.cuni.cz (L.Č.)

[2] Department of Pharmaceutical Technology, Faculty of Pharmacy in Hradec Králové, Charles University, 50005 Hradec Králové, Czech Republic; holao3aa@faf.cuni.cz

* Correspondence: vokral@faf.cuni.cz

Absorptive and secretory permeability of digoxin in Caco-2 cells

Table S1. Effects of the antiretrovirals and the model inhibitor CP100356 on ABCB1-controlled [^{3}H]-digoxin transport across Caco-2 monolayers—Papp values.

Compound	Concentration	$P_{app\ A\ to\ B}$[1] (10^{-6} cm s^{-1})	$P_{app\ B\ to\ A}$[2] (10^{-6} cm s^{-1})
Control	6 nM	1.08 ± 0.53	9.48 ± 3.82
+ CP100356	2 μM	4.74 ± 0.41 ***	6.69 ± 0.07
+ Abacavir	100 μM	1.77 ± 0.27	18.02 ± 1.33 **
+ Atazanavir [3]	20 μM	1.96 ± 0.39	10.69 ± 0.71
	50 μM	3.03 ± 0.90 ***	3.39 ± 0.90 *
+ Darunavir	20 μM	2.73 ± 0.48 *	16.45 ± 2.05 *
	50 μM	2.65 ± 1.11 **	8.85 ± 4.33
	100 μM	3.65 ± 0.52 ***	6.26 ± 0.06
+ Dolutegravir [4]	10 μM	2.06 ± 0.02	24.54 ± 4.49 ***
+ Etravirine [4]	20 μM	1.14 ± 0.20	3.66 ± 0.17
+ Lopinavir [3]	5 μM	2.06 ± 0.81	11.65 ± 7.06
	50 μM	3.40 ± 0.74 ***	6.44 ± 1.01
+ Maraviroc	20 μM	1.39 ± 0.31	15.59 ± 3.67
	100 μM	1.74 ± 0.03	15.34 ± 2.48
+ Rilpivirine [3]	20 μM	3.37 ± 0.64 ***	4.93 ± 1.09
+ Ritonavir [3]	20 μM	2.32 ± 0.69	6.17 ± 1.75
	50 μM	3.24 ± 0.45 ***	3.62 ± 0.79 *
+ Saquinavir [3]	5 μM	2.19 ± 0.86	17.17 ± 0.07 *
	20 μM	3.67 ± 1.90 ***	4.88 ± 1.71
+ Tenofovir DF	100 μM	0.97 ± 0.05	11.36 ± 0.63
+ Zidovudine	100 μM	0.53 ± 0.06	7.08 ± 0.70

Statistical analysis was performed using the ordinary one-way ANOVA with Dunnett's post hoc multiple comparisons test. Values differing significantly from the control are indicated by the labels * (p <0.05), ** (p < 0.01), or *** (p < 0.001).

[1] $P_{app\ A\ to\ B}$, absorptive permeability.

[2] $P_{app\ B\ to\ A}$, secretory permeability.

[3] Higher concentrations were not tested because a rPapp of approximately 1 was reached.

[4] Higher concentrations were not tested due to limited solubility.

Table S2. Effects of the tested DAAs and the model inhibitor CP100356 on ABCB1-controlled [^{3}H]-digoxin transport across Caco-2 monolayers—Papp values.

Compound	Concentration	$P_{app\ A\ to\ B}$[1] (10^{-6} cm s^{-1})	$P_{app\ B\ to\ A}$[2] (10^{-6} cm s^{-1})
Control	6 nM	1.08 ± 0.53	9.48 ± 3.82
+ CP100356	2 µM	4.74 ± 0.41	6.69 ± 0.07
+ Asunaprevir [3]	20 µM	3.28 ± 0.43 ***	10.21 ± 3.03
	50 µM	3.93 ± 0.10 ***	4.96 ± 0.58
+ Daclatasvir [3]	5 µM	1.51 ± 0.51	14.63 ± 4.32
	20 µM	2.45 ± 0.12 *	2.96 ± 0.66 *
+ Elbasvir [4]	5 µM	3.48 ± 0.30 ***	20.30 ± 1.75 ***
+ Grazoprevir [3]	20 µM	2.74 ± 0.62 *	10.29 ± 1.61
	50 µM	4.13 ± 0.75 ***	4.91 ± 0.29
+ Ledipasvir [4]	20 µM	1.65 ± 0.13	15.53 ± 3.37 *
	50 µM	1.71 ± 0.92	6.35 ± 2.67
+ Sofosbuvir	100 µM	1.63 ± 0.25	9.94 ± 1.12
+ Velpatasvir [4]	5 µM	3.41 ± 0.48 ***	24.75 ± 2.66 ***

Statistical analysis was performed using the ordinary one-way ANOVA with Dunnett's post hoc multiple comparisons test. Values differing significantly from the control are indicated by the labels * (p <0.05) or *** (p < 0.001).

[1] $P_{app\ A\ to\ B}$, absorptive permeability.

[2] $P_{app\ B\ to\ A}$, secretory permeability.

[3] Higher concentrations were not tested because a rPapp of approximately 1 was reached.

[4] Higher concentrations were not tested due to limited solubility.

Docking of selected antivirals binding to ABCB1—methodology

The mouse ABCB1a crystal structure (PDB code: 4M1M) was selected as the starting geometry for the receptor structure [84]. Non-conserved and distant water molecules were removed from the structure. All subsequent operations were performed using Molecular Operating Environment (MOE, version 2020.09). The PDB structure was diagnosed using the structure preparation panel and structural issues (namely incorrect residue types, missing residues, and protein chain C- or N-termini that needed to be charged or capped) were fixed using the MOE correction protocol. Hydrogens were then added at positions appropriate for pH 7 using the Protonation 3D protocol and partial charges were assigned to the whole protein structure using the AMBER 94 forcefield. The strain energy of the complete resulting system was minimized using a gradient of 0.001 RMS kcal/mol/Å2. The ligand binding domain (LBD) was identified and characterized using MOE's Site Finder and dummy atoms were placed at alpha centers. The LBD characterized in this study is very similar to that described by Bocci et al. [20].

Ligand molecules were prepared using the MOE Builder interface by adding partial charges and performing energy minimization of their structures. Docking of the known ligands RHD123 and digoxin as well as novel molecules was performed using Alpha PMI placement (samples per conformation: 20; no. of poses: 250). Dummy atoms assigned by Site Finder were used as sites for placing molecules. The London dG scoring function was used to assess the free binding energy of the placed molecules in each pose. Poses of molecules within the LBD were then refined using the rigid receptor setup. The free binding energies of the final poses were scored using the GBVI/BSA dG scoring function.

Docking of selected antivirals binding to ABCB1—results

Docking experiments were performed to support the experimental studies. Due to the absence of a human ABCB1 crystal structure, we used the crystal structure of mouse ABCB1a instead. After identifying the binding cavities for RHD123 and digoxin, we performed docking simulations with all of the other tested compounds to compare their free energies of binding and identify the highest scoring poses within the cavity. The binding free energies of each studied compound are listed in Table S3.

Table S3: Binding free energies of the top scoring poses of RHD123, digoxin, CP100356, and the studied antivirals in the ABCB1a mouse crystal structure.

Compound	Binding Free Energy(kcal/mol)
RHD123	-6.74
Digoxin	-8.55
CP100356	-8.30
Atazanavir	-9.16
Abacavir	-6.89
Daclatasvir	-9.90
Dolutegrasvir	-7.64
Elbasvir	-7.76
Etravirine	-8.18
Lopinavir	-10.18
Maraviroc	-8.85
Rilpivirine	-7.48
Ritonavir	-10.29
Sofobusvir	-8.49
Tenofovir DF	-8.60

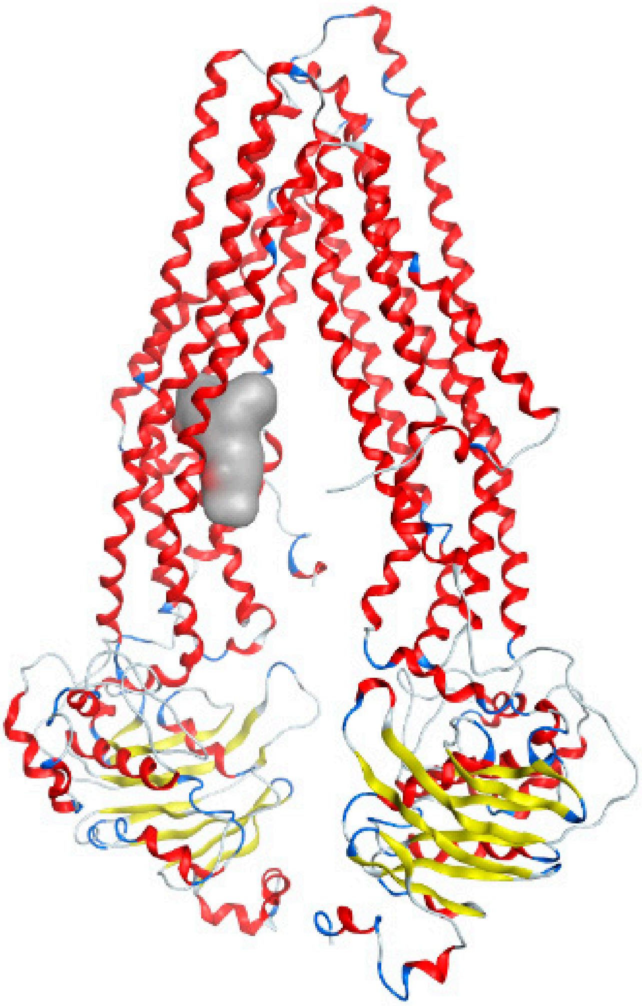

Figure S1. Location of the binding cavity used in the docking experiment within the ABCB1a crystal structure (PDB code: 4M1M). The cavity was identified and rendered using MOE's Site Finder tool.

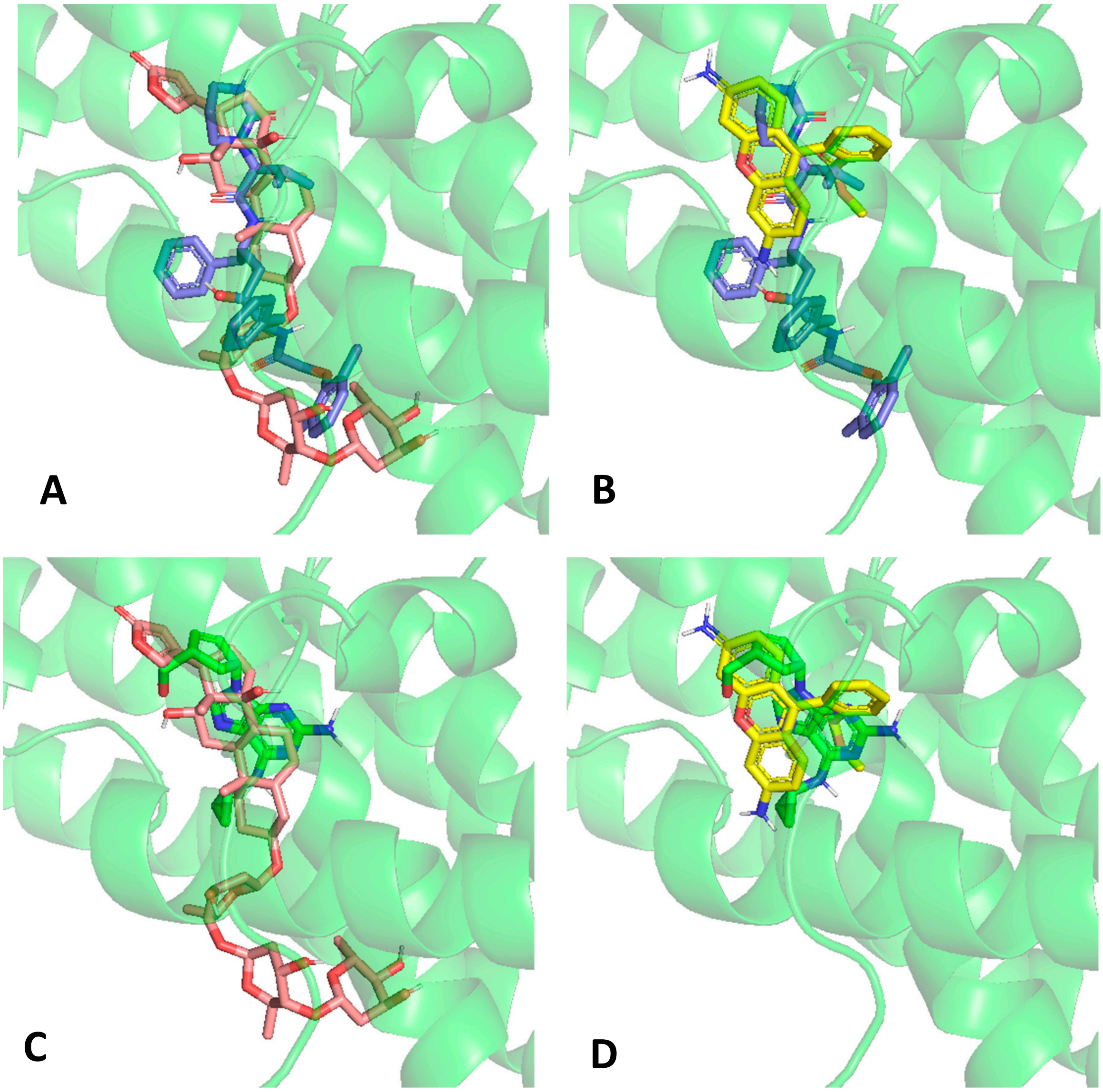

Figure S2. Visualizations of the top scoring poses of lopinavir (purple; a strong inhibitor of digoxin and RHD123 transport) and abacavir (green; a drug with no effect on digoxin and RHD132 transport) within the ligand binding cavity. (**A**) lopinavir + digoxin (pink), (**B**) lopinavir + RHD123 (yellow), (**C**) abacavir + digoxin, (**D**) abacavir + RHD123.

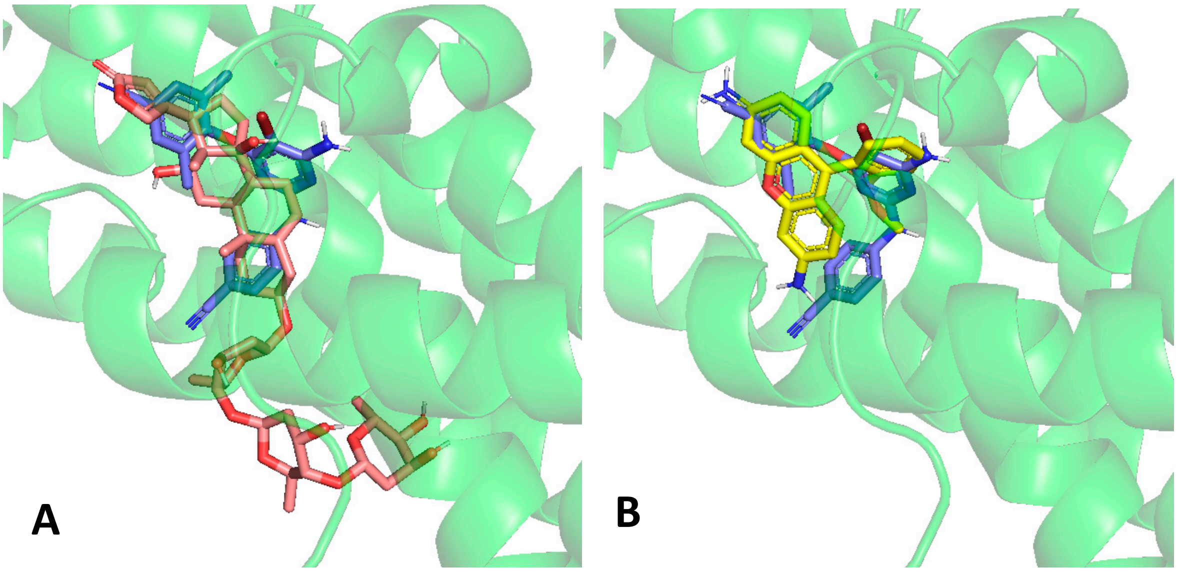

Figure S3. Visualizations of the top scoring pose of etravirine (purple) within the ligand binding cavity together with (**A**) digoxin (pink) and (**B**) RHD123 (yellow). Etravirine inhibited the transport of digoxin but not RHD123 in the Caco-2 model.

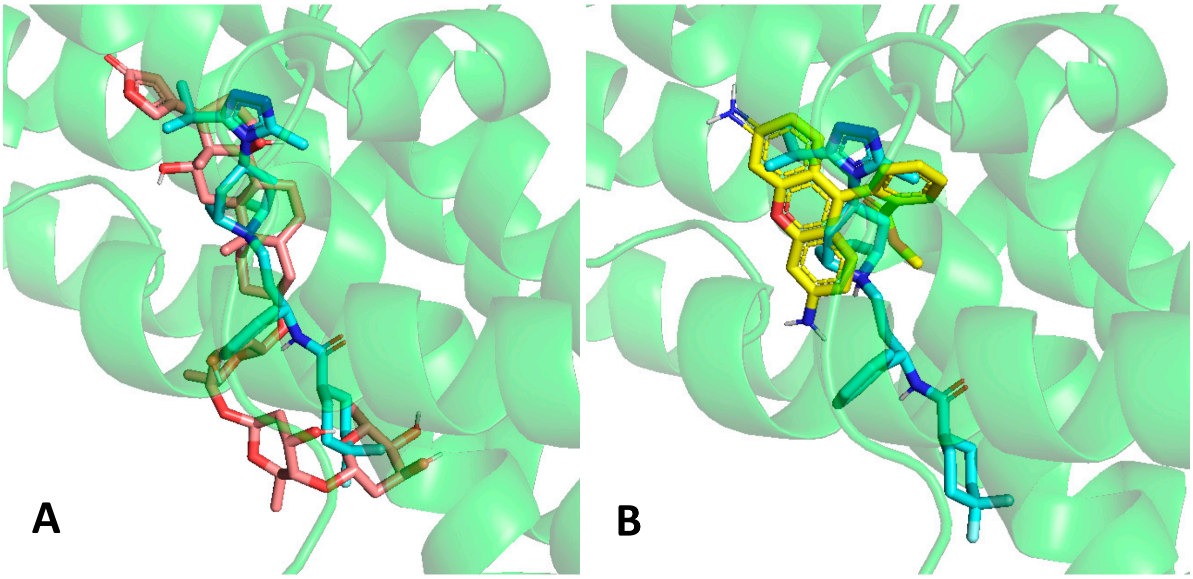

Figure S4. Visualizations of the top scoring pose of maraviroc (turquoise) within the ligand binding cavity together with (**A**) digoxin (pink) and (**B**) RHD123 (yellow). Maraviroc inhibited the transport of RHD123 but not digoxin in both experimental models.
